# Construction of Genomic Library and Screening of *Edwardsiella tarda* Immunogenic Proteins for Their Protective Efficacy Against Edwardsiellosis

**DOI:** 10.3389/fimmu.2021.764662

**Published:** 2021-11-16

**Authors:** Palanisamy Bothammal, Mohan Ganesh, Vellaisamy Vigneshwaran, Kumarasamy Anbarasu, Karuppiah Ponmurugan, Naif Abdullah Al-Dhabi, Kalimuthusamy Natarajaseenivasan

**Affiliations:** ^1^ Medical Microbiology Laboratory, Department of Microbiology, Center for Excellence in Life Sciences, Bharathidasan University, Tiruchirappalli, India; ^2^ Microbial Biotechnology Laboratory, Department of Marine Biotechnology, School of Marine Sciences, Bharathidasan University, Tiruchirappalli, India; ^3^ Department of Botany & Microbiology, College of Science, King Saud University, Riyadh, Saudi Arabia

**Keywords:** *Edwardsiella tarda*, edwardsiellosis, immunoreactive protein, vaccine, IL-10, IFN-γ

## Abstract

*Edwardsiella tarda* is a severe aquaculture pathogen that can infect many hosts including humans, animals, and fish. Timely diagnosis and treatment are crucial for the control of edwardsiellosis in the aqua industry. By using rabbit polyclonal antibody, an expression gene library of virulent *Edwardsiella tarda* strain ED-BDU 1 isolated in south India was constructed and screened. The identified immune expressive proteins were characterized, and the corresponding coding sequences were cloned, expressed, and the purified recombinant proteins were used as antigens. The identified immunoreactive proteins namely HflC, HflK, and YhcI were studied for their immune protective potential *in vivo* by challenge experiments. The protective efficacy of HflC, HflK, and YhcI showed that the clearance of *Edwardsiella* from the host with ~ 60% survivability. Further, the immunoreactive proteins induce a strong immune response upon infection and elicit the significant production of IL-10, IFN-γ, Th1, and Th2 mediated mRNA expression and were therefore effective in vaccine production for edwardsiellosis.

## Introduction

Aquaculture is a fast-growing fisheries sector globally, and India accounts second in culture fisheries production with an annual growth rate of over 7%. Aquaculture not only supplies dietary essentials for human consumption but provides opportunities for employment and income, especially in less economically developed rural areas. Sixty million people are directly engaged, part-time or full-time, in the primary production of fish, either by fishing or in aquaculture, supporting the livelihoods of 10-12% of the world population. It currently accounts for over 50% of global fish consumption. In India, carp varieties are highly cultivated, namely catla (*Catla catla*), rohu (*Labeo rohita*), and mrigal (*Cirrhinus mrigala*) (1).

The aquaculture industry has become increasingly vulnerable to exotic, endemic, and emerging disease epizootics. It is estimated that as much as 10% of all aquatic aquaculture animals die of infectious diseases, representing losses of more than USD 10 billion per year globally. The most prevalent bacterial pathogens in Indian aquaculture belong to the genera *Aeromonas, Edwardsiella, Vibrio*, and *Flavobacterium*, infecting the top farmed fish species (2–4). The genus *Edwardsiella* belongs to the family *Enterobacteriaceae* and consists of three known species, namely *E. tarda, E. ictaluri, E. hoshinae * (5). The Gram-negative *E. tarda*, ubiquitous in the microbial biosphere and has the broadest host range, infecting both freshwater and marine life. Fish infected by *E. tarda* develop edwardsiellosis, generalized septicemia with symptoms including distended abdomen, prolapsed rectum, and gross lesions of internal organs (2). Clinical presentation of edwardsiellosis such as extensive skin lesions that progress into necrotic abscesses, distended abdomen, and swollen anus due to the accumulation of ascetic fluid, pigment loss, enlarged kidney, and abscesses on internal organs.

Virulence factors of *Edwardsiella* help entry to host fish through the gastrointestinal tract, the gills, and the body surface and can resist the host immune system mediated by host complements and phagocytes. *E. tarda* uses various secretion systems as virulence factors likely type III (T3SS) and type VI (T6SS) secretion systems, which are vital to invasion and intracellular replication of microorganisms in the host (6–9). Apart from that, the production of enzymes such as hemolysins and chondroitinase helps the bacterium to invade inside the host (10, 11). *In vivo* induced antigen technology (IVIAT) used examined the biological properties and function of a putative adhesin, Eta1, which is drastically enhanced during infection of host cells (12). Thus, studies of *in vivo* expressed proteins contribute to the understanding of host-pathogen interactions and are helpful in the design of novel diagnostics and vaccines.

The outbreak of edwardsiellosis causes major economic losses in cultured fish. Currently, the control of edwardsiellosis relies chiefly on the use of antibiotics. The use of antibiotics poses the risk of selection of drug resistance in pathogens, making the treatment ineffective, and risking the spread of resistance determinants to other bacteria (13). Pathogenic *E. tarda* is found to be an intracellular pathogen and is naturally resistant to benzylpenicillin, oxacillin, macrolide, lincosamides, streptogramins, and glycopeptides which increases the difficulty of antibiotic-based treatment and their application in aquaculture has become more and more restricted (14, 15). It is, therefore, urgent to develop new prevention and treatment methods.

Routinely using antibiotics in aquaculture leads to the emergence of multi-drug resistant pathogens in aquaculture, in order to avoid such multi drug resistance and spreading of infection vaccination would be best the eco-friendly practical approach. Several vaccines are reported so far against edwardsiellosis. Immunization using FKC or LPS showed protective effects after challenge with a virulent strain of *E. tarda* in Japanese eel  (16). Kawai et al (17), reported ghost cells and bacterin of *E. tarda*, showing that this was most effective (relative percent of survival rate above 90%). Using purified recombinant subunit vaccine as antigen is more immunoprotective. Recombinant vaccines such as OmpA and Omp48 showed high levels of immunogenicity against edwardsiellosis (18). The main advantage of recombinant vaccines is that they are safer because they only contain the antigenic protein and not the entire pathogens, and these genetically engineered vaccines help remove undesired harmful antigens.

In the present study, we have identified *in vivo*-expressed proteins of virulent *Edwardsiella tarda* ED-BDU 1 through an expression gene library screening. The library was screened with *Edwardsiella tarda* specific rabbit polyclonal sera, and the identified proteins were evaluated for their protective efficacy for edwardsiellosis.

## Materials and Methods

### Bacterial Strains, Growth Conditions, and Ethics

*E. coli* BL21, BL21 (DE3), and DH5α were purchased from Novagen (Novagen Inc., Madison, Wisconsin). *E. tarda* BDU-1 was isolated from the kidneys of diseased *Labeo rohita* and is naturally resistant to rifampin. All strains and isolates were grown in Luria-Bertani broth (LB) (28) at 37°C (for *E. coli*) or 28°C (for *E. tarda* ED-BDU1).

Animal experiments described in this study were carried out in strict accordance with the recommendations approved by the Committee for the Purpose of Control and Supervision on Experiments on Animals (CPCSEA), and Bharathidasan University Ethics Committee in Animal Experimentation (Approval number: BDU/IAEC/2011/29).

### Study Area and Collection of Diseased Fish

The present study was conducted in and around Tiruchirappalli district, Tamil Nadu. A total of 41 farms were included in the study. Of the 41 fish farms, 10 were reported to have a high mortality rate among farmed fishes. The infected fishes were collected and transported on ice to the Medical Microbiology Laboratory on the same day of collection. Information data sheets comprising the clinical sign and mortality rate of fish were collected with duly signed by the fish farm proprietor.

### Antibiotic Susceptibility Test

All the isolates were subjected to the antibiotic susceptibility test by Kirby-Bauer disk diffusion method by using Muller-Hinton Agar (Merck, Germany). Briefly, the sample of *E. tarda* culture in Tryptic soy broth was swabbed onto Muller Hinton agar uniformly for a lawn of bacterial growth. Antibiotic discs were gently placed on the surface of the agar using sterile forceps and were kept in the incubator for 24h at 30°C. Interpretation of the resulted inhibition zones, namely sensitivity and resistance, was done according to the standard measurement in millimeter (mm) following National Committee for Clinical Laboratory Standards.

### Phenotypic and Genotypic Characterization

The *E. tarda* isolates from diseased fishes were characterized by morphological and biochemical tests. The surface of the fish was washed, and visceral organs were removed aseptically. The organs were homogenized, serially diluted in PBS, and plated on Rimler-Shotts medium. Isolates identified as *E. tarda* by biochemical test and further confirmed by molecular characterization of the 16S rRNA sequencing using specific 16S-F 5’-AGAGTTTGAT(C/T)(A/C)TGGCTCAG-3’ and 16S-R 5’- AAGGAGGTGATCCAG -3’ primers. The temperature profile for amplification was initial denaturation at 95°C for 4 min, denaturation at 95°C for 30 sec, annealing at 55°C for 45 sec, and extension at 72°C for 1 min, for 30 cycles, followed by a final extension at 72°C for 5 min. The PCR products were purified, sequenced, and analyzed for phylogenetic relatedness to strains from type culture collections.

### Enzyme-Linked Immunosorbent Assay

Two young healthy rabbits weighing 3kg were selected and their sera were pretested for *E. tarda* antibodies prior to inoculation. Live antigens were prepared from 24 hours old *E. tarda* ED-BDU1 culture (1-2 X 10^8^/ml). The cultures were centrifuged at 10,000 x g for 10 mins and dissolved in Phosphate buffered saline (PBS) and the cells count was determined by using a Neubauer counting chamber. Briefly, 1-2 x 10^8^ cells were injected subcutaneously into the rabbits on day 1, followed by booster inoculums on days 14 and 28. The level of antigen-specific circulating antibodies was determined by titrating the serum samples in ELISA. For performing serum dilution in ELISA, wells were coated with 100μl carbonate coating buffer (pH 7.2) containing 1μg of sonicated antigen and stored at 4⁰C overnight. Then the plate was proceeding to wash with phosphate-buffered saline with Tween 20 (PBST) (8mM Na_2_HPO_4_, 150mM NaCl, 2mM KH_2_PO_4_, 3mM KCl, 0.05% Tween^®^ 20, pH 7.4) and blocked with 200μl/well of 4% non-fat milk in PBST for 1 h at 37°C. Sera were diluted from starting dilution of 1:25 to 1:51200 in triplicate and incubated for 1 h at 37°C. Bound IgG was detected using peroxidase-conjugated Protein A (Sigma, St. Louis, MO), followed by development using 100μl of ortho phenylenediamine (OPD). Optical densities (OD’s) were read at 490nm using an ELISA reader (Bio-Rad, Hercules, CA, USA).

### Genomic DNA Library Construction and Screening

Among all the isolates *E. tarda* ED-BDU1 was found to be highly resistant, so we constructed a genomic DNA expression library for our local isolate. *E. tarda* strain ED-BDU1 was constructed using λZAPII library (Strategene, San Diego, CA). Briefly 5 x 10^4^ pfu of library construct was used for amplification. Validation of the entire library was carried out by PCR. A λZAPII library consisting of 3- to 5-kb random fragments of *E. tarda* ED–BDU1 was screened to identify phage that expressed gene products reactive with rabbit polyclonal antibodies specific for *E. tarda*. The secondary antibody was peroxidase-conjugated Protein A (Sigma, St. Louis, MO) diluted 1:2000. After primary and secondary antibody treatment, the NC membrane was developed with the chromogenic substrate 4-chloro-1-napthol (Sigma, St. Louis, MO).

### *In Vivo* Excision of Immunoreactive Clones and DNA Sequencing

Excision of *in vivo* expressed clones was performed by the previously reported method with slight modifications (19). Suspected plaques on agar plugs were transferred to 500 µl of SM buffer and allowed to elute overnight. Insert-carrying plasmids were rescued from selective reactive phages by using ExAssist helper phage and *E. coli* SOLR according to the manufacturer’s instructions. Plasmid DNA was isolated from reactive clones using a QIAprep spin miniprep kit (Qiagen, Valencia, CA) and sequenced in a commercial sequencing facility (Macrogen, South Korea) using T3, T7, and custom-designed primers. Sequences were edited with Chromas 1.61 (Technelysium Pty. Ltd., Queensland, Australia) and aligned and connected with DNASIS (Hitachi Software Engineering Co., Ltd., San Francisco, CA). DNA sequences thus obtained were compared with the whole-genome sequences of *E. tarda* strain FL95-01 and *E. tarda* strain ET-1 using the National Centre for Biotechnology Information server (https://blast.ncbi.nlm.nih.gov/Blast.cgi) and highly identical sequences were retrieved.

### Cloning

The specific primers used in this study were listed in **Table 1**. The sequence of respective genes was PCR amplified from genomic DNA of ED-BDU1, which was denatured initially at 94°C for 5 mins followed by 30 cycles of 94°C for 1 min, 57°C for 45 sec and 72°C for 1 min and 72°C for 7 mins. Following PCR amplification, the amplicons were cloned into predigested pET28a (Novagen, Madison, WIS) (*hflC, hflK*, and *yhcI*). Recombinant plasmids were transformed into *E. coli* BL21 (DE3). Colony PCR, restriction endonuclease analysis (REA), and DNA sequencing were performed to confirm the presence of the correct insert.

**Table 1:** List of primers used for amplification of hflC, hflK, and yhcI genes.

Gene name	Sequence
hflC- F	5’- CCGGAATTCATGCGTAAGTCTTTGTTAGTGATTC-3’
hflC- R	5’- CCCAAGCTTTTACTTTGCGCTTTTGCCG-3’
hflK- F	5’-CGGAATTCATGGCGTGGAATCAGCCC-3’
hflK- R	5’-CCCAAGCTTTTATTGCTCTTGTCATCCACCAG-3’
yhcI- F	5’- CGCGGATCCATGAATACGCTGGCCATT -3’
yhcI- R	5’-GTGCTCGAGTCATGCCGTTTCTCTCC -3’

### Colony PCR

The construct was extracted from the transformed cells by the boiling method. Briefly, each single colony was picked separately and dissolved in MilliQ water. The content was boiled for 8 mins in a water bath and immediately kept in ice for 2 min. The vial was centrifuged at 9000 rpm for 10 min and the supernatant was directly used as a template for the colony PCR. Amplification was performed in a thermal cycler (Eppendorf, Germany) at 94°C for 5 min, followed by 30 cycles of 94°C for 1 min, 69°C for 45 s, and 72°C for 1 min, with a final single extension of 72°C for 7 min. The presence and absence of amplicons in the corresponding colonies were confirmed by agarose gel electrophoresis.

### Restriction Endonuclease Analysis

The positive colony was picked and streaked into the fresh LB agar plate with kanamycin as a resistant marker. Once the desired growth was attained, a single colony was inoculated into the 5 ml of LB broth containing appropriate antibiotic and incubated 37°C for 12-16 hrs. The construct was extracted by using a mini plasmid extraction procedure (Qiagen mini preparation kit) as described above. The restriction analysis was carried out as follows, 5 ul of universal fast digest buffer (33mm Tris Acetate, 10 mM Magnesium Acetate, 66mM Potassium Acetate and 0.1 mg/ml BSA), 1 µl of Hind III and 1 µl of EcoRI, 1µl of BamHI, 1 µl of XhoI, 10 µl of construct (hflc + pET-28a, hflk + pET-28a, yhc1 + pET-28a) and 33 µl of Milli-Q. The digestion was carried out at 37°C for 1 hour. Agarose gel electrophoresis was carried out with empty pEt-28a, construct single and double digest and finally amplified product of *hflc*, *hflk*, and *yhc1* in ascending order with molecular markers on both sides of the gel.

### Expression and Purification

Recombinant plasmid pET28a+hflC, pET28a+hflK, and pET28a+yhcI were transformed into *E. coli* BL21 (DE3). Briefly, the recombinant cells were cultured in 500 mL of LB broth containing antibiotic Kanamycin 3 mg/ml with proper agitation. When cultures reached an optical density of 0.6 at 600 nm 1mM IPTG was added to the culture to induce the expression of polyhistidine-tagged recombinant proteins (HflC, HflK, and YhcI) and cells were harvested after 3h. Cultures measuring 500mL were pelleted by centrifugation at 10,000 rpm for 10 mins at 4°C in a falcon tube and the pellets were resuspended with 1:10 (w/v) of lysis buffer (50 mM sodium phosphate, 300 mM NaCl, 2.5mM imidazole, 8M urea, 200 μg/ml of lysozyme, and 2 mM phenylmethylsulfonyl fluoride [PMSF] pH 8.0). Cells were lysed by sonication five times at setting 3 for 10 seconds each time, with cooling on ice for 1 minute between each sonication. Cell extract was centrifuged at 10,000 x g for 30 minutes at 4°C in a high-speed cooling centrifuge. Recombinant His-tagged proteins (HflC, HflK, and YhcI) were purified by using IMAC Ni2+ resin (Bio-Rad, USA) in a buffer containing 8M urea. Then 500 µl cell lysate supernatant was added onto a 5 mL nickel column equilibrated with 0.5 ml binding buffer (20 mM sodium phosphate, 300 mM NaCl, 5 mM imidazole, and 8M urea, pH 8.0). It was incubated in ice for 5 minutes and centrifuged at 1000 x g for 1 minute at 4°C in a high-speed cooling centrifuge. The column was washed with the addition of 0.5 ml of wash buffer (20 Mm sodium phosphate, 300 mM NaCl, 10 mM imidazole, and 8M urea, pH 8.0). The elution was performed for the target by adding a five-column volume of elution buffer (50 mM sodium phosphate, 300 mM NaCl, 250 mM Imidazole, and 8M urea pH 8.0), was added onto the column, mixed well, and incubated for 5 mins to elute the His-tagged protein. The concentration of purified recombinant protein was determined using a Bicinchoninic acid assay kit and measured at 562 nm in the biophotometer.

### SDS-Polyacrylamide Gel Electrophoresis and Western Blotting

Proteins were separated on 10% acrylamide gels by SDS-PAGE and transferred electrophoretically to a nitrocellulose membrane. After blocking, membranes were incubated with rabbit polyclonal antibodies followed by incubation with anti-human or anti-rabbit IgG conjugated with horseradish peroxidase (Sigma, St. Louis, MO). Membranes were developed and visualized using SuperSignal™ West Pico PLUS Chemiluminescent Substrate (Thermo Scientific, Waltham, Massachusetts, United States). All incubations were performed at room temperature and TBST washing was done thrice each for 10 min after every incubation. The NC membranes were documented with a Fusion Solo 6S (VilberLourmat, Collegien, France).

### *Edwardsiella* Bacterins and Recombinant Proteins

*Edwardsiella* bacterins, whole-cell lysate (WCL) was prepared from *E. tarda* strain ED-BDU1. For vaccine preparation and immunization, ED-BDU1 cells with an OD of 1.0 were harvested by centrifugation at 4,000 g for 15 min and washed three times with saline solution. Washed cells were suspended in sterile saline solution as a live *E. tarda* vaccine or then incubated at 100°C to produce the inactivated vaccines. Plate counting assay was performed to examine bacterial sterility in the inactivated vaccines. HflC, HflK, and YhcI recombinant proteins were over-expressed and purified using the *E. coli* expression system as described above. The recombinant proteins were dialyzed, and the protein concentration was determined using a Bicinchoninic acid assay (BCA) kit (Sigma, St. Louis, MO). Then 100 µg of protein in Alhydrogel (Sigma, St. Louis, MO) was used as a vaccine formulation for the first booster on day 0. The second booster of 100 µg protein was administered by subcutaneous injections on the 21^st^ day.

### Immunization and Challenge Experiments

Healthy Indian common carp (*L. rohita*, weighing 80-90 g) were purchased from the SDS Fish Farm, Tanjore District, Tamil Nadu, India. The fish were acclimatized in the laboratory for two weeks before experimental manipulation. Fish were maintained in aerated water and fed daily with commercial dry pellets. Before each of the immunization experiments, the fish were anesthetized with tricaine methanesulfonate (TMS, Sigma) prior to immunization experiments.

Grouping of mice: Laboratory mice were divided randomly into the following six groups, each group containing five mice (i) First group, positive control, were injected with heat-killed whole cell lysate; ii) Second group, negative control, were injected with PBS alone; iii) Third group, internal control, were injected with adjuvant (Alhydrogel (Sigma, St. Louis, MO)) alone; iv) Fourth group mice were injected with HflC recombinant protein mixed with adjuvant; v) Fifth group mice were injected with HflK recombinant protein mixed with adjuvant; and vi) the Sixth group of mice were injected with YhcI recombinant protein mixed with adjuvant.

Grouping of fishes: *L. rohita* were divided randomly into the following six groups, each group containing ten fish. (i) Positive control fish were injected with heat-killed whole cell lysate, ii) Negative control fish were injected with PBS alone, iii) Internal control fish were injected with adjuvant [Alhydrogel (Sigma, St. Louis, MO)] alone, iv) group four fish were injected HflC recombinant protein mixed with adjuvant, v) group five fish were injected HflK recombinant protein mixed with adjuvant, vi) and group six fish were injected YhcI recombinant protein mixed with adjuvant.

In brief, the recombinant proteins were mixed with an equal volume of complete adjuvant and injected into the animals on day 1, and followed by 2 booster doses which were injected with recombinant protein mixed with an equal volume of incomplete adjuvant at days 21 and 35. For each dose 10µg of recombinant proteins were injected into the respective group. The positive control was injected with heat-killed whole cell *Edwardsiella*, as a negative control animals received PBS.

The animals were challenged intraperitoneally with 5x median lethal dose (LD50) of *E. tarda* ED-BDU1 (4 x 10^7^ CFU) 42 days after the first immunization. Animals were monitored daily and those who survived were euthanized on day 70 after the challenge. Similarly, pathogen-free 4-6 weeks old female BALB/c mice with a weight of 20–35 g were used for immunization and challenge experiments. Animals were bled through the tail vein before each immunization on day 0 (pre-vaccination), 14th (after 1st immunization), 28th (after booster), 42nd (after booster), 49th and 70th day (after challenge), and serum was collected and kept at -80°C until use.

### Humoral Immune Response

Humoral immune response was determined using ELISA as mentioned before. In Brief, 0.2 µg/well of rHflC, rHflK, and rYhcI were coated in polystyrene 96 well microtiter plates (Nunc; Thermo Scientifics, USA) using carbonate coating buffer (pH 9.6) and incubated overnight at 4°C. The plate was blocked with 4% non-fat milk in PBST. Mice sera (1:200) dilution in PBST were added and incubated for 1h at 37°C. Anti-mouse IgG peroxidase conjugate (Sigma Aldrich, St. Louis, MO) (1:4000) dilution in PBST was added and incubated at 37°C

The reaction was developed with o-phenylenediaminedi]hydrochloride (Sigma-Aldrich, St. Louis, MO). After 5 mins dark incubation the reaction was stopped by the addition of 0.1M sulphuric acid (Merk, Germany). Optical density (OD) values were measured at 490 nm (Bio-Rad, Hercules, CA, USA).

### Cell-Mediated Immune Response

Cell-mediated immune responses were analyzed by cytokine profiling of immunized mice spleen cells. Briefly, total RNA was isolated from the immunized mice spleen cells using the TRIzol reagent (Invitrogen, Carlsbad, CA). Using the iScript cDNA synthesis kit the cDNA was synthesized (Bio-Rad, Hercules, CA, USA). A 25 μL reaction mixture containing 50 ng cDNA, 12.5μL Master Mix (SYBR Green PCR Master Mix (Bio-Rad, Hercules, CA, USA)), 0.5μM of each primer) was used, the amplification process performed in CFX96 TouchTM Real-Time PCR detection system (Bio-Rad, Hercules, CA, USA). All the primers used in this experiment are given in **Table 2**. The amplification process consisted of 95°C for 10 min for initial denaturation, followed by for 45 cycles of 95°C for 5 s, 60°C for 30 s, and a variable extension time at 72°C. After amplification, the melting curves were analyzed at a linear temperature transition rate of 0.1°C/s from 55 to 95°C, with continuous fluorescence acquisition.

**Table 2:** List of cytokine primers used for qPCR analysis.

Gene Name	Direction	Sequence (5’- 3’)
TNFα	Forward	GGACTAGCCAGGAGGGAGAA
	Reverse	CGCGGATCATGCTTTCTGTG
IL 10	Forward	GCCCTTTGCTATGGTGTCCT
	Reverse	TTTTCAGGGATGAAGCGGCT
IL 4	Forward	CAAACGTCCTCACAGCAACG
	Reverse	AAGCCCGAAAGAGTCTCTGC
IL 12p40	Forward	GGAAGCACGGCAGCAGAATA
	Reverse	AACTTGAGGGAGAAGTAGGAATGG
IFNγ	Forward	ATTCAGAGCTGCAGTGACCC
	Reverse	GGAAGCACCAGGTGTCAAGT
GAPDH	Forward	AACGACCCCTTCATTGAC
	Reverse	TCCACGACATACTCAGCAC

### Statistical Analysis

All data were normally distributed and presented as mean values ± SEM. In the case of single mean comparisons, data were analyzed by Student’s t-test or, when not normally distributed a nonparametric Mann-Whitney U test. Differences in means among multiple data sets were analyzed using 1-way ANOVA/2-way ANOVA with Tukey test. P values less than 0.05 were found to be significant. All the data were computed with GraphPad Prism 8 or SigmaPlot 11.0 Software.

## Results

### Identification and Characterization of *E. tarda*

**Figure 1:**
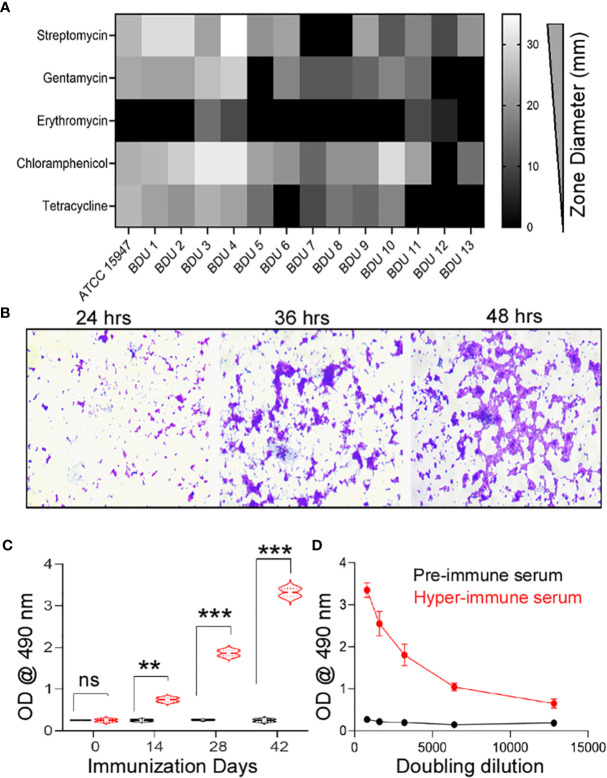
**(A)** Antimicrobial Resistance of the *E. tarda* ATCC 15947 and the clinical isolates. x-axis: Antimicrobial zone in millimeter, y-axis: different antimicrobial agents. **(B)** Biofilm forming ability of the strain *E. tarda* ED-BDU1 at different time intervals (400X). **(C)** Immune response in rabbits against the live antigen of ED-BDU1 at different days of immunization. X-axis- Immunization days, Y-axis- OD @ 490 nm, ns, no significance, **P < 0.01, ***P < 0.001. **(D)** Doubling dilution of the polyclonal rabbit antisera raised against the live antigen of ED-BDU1, X-axis- Doubling dilution of the sera taken after 42 days of immunization, Y-axis- OD @ 490 nm.

In 10 different high mortality fish farms (**Table S1**), 27 infected Indian common carp (*Labeo rohita*) exhibited clinical manifestations such as gross lesions on the skin, pale gills, and tumefaction of the eye. A total of 54 isolates were obtained from 27 infected fishes with an isolation frequency of two isolates per infected fish. The isolates thus obtained were sub-cultured until they reached clonality. The morphological and biochemical studies identified 13 isolates (24.1%) as *E. tarda* (**Table S2**), 11 (20.4%) as *Salmonella* sp., and 10 (18.5%) as *Shigella* sp. All the isolates were found to be resistant to penicillin (piperacillin, oxacillin, ampicillin), aminoglycoside (neomycin), sulfonamides (sulfamethoxazole), peptide (polymyxin), macrolide (oleandomycin, erythromycin), and lincosamide (lincomycin) groups of antibiotics. Intermediate resistance was observed for tetracyclines (tetracycline; 100%), aminoglycosides (gentamycin, 75% and streptomycin, 95%), and other (chloramphenicol, 90%) groups of antibiotics (**Figure 1A**). The strain *E. tarda* ED–BDU1 showed excellent biofilm-forming ability apart from antibiotic resistance (**Figure 1B**). **Figure S1A** represents the genomic DNA of the *E. tarda* isolates on a 0.8% agarose gel stained with ethidium bromide. Further, the size of the 16S rRNA product of *E. tarda* was found to be 1537 bp (**Figure S1B**). Then the isolates shared 93-96% homology to known *E. tarda* isolates. Three sequences were deposited in NCBI GenBank (Accession no: KT001505, KT001506, KT001507).

### Rabbit Polyclonal Antibody Against *E. tarda*

The immunized sera from rabbits presented significant levels of circulating antibodies at 14, 28, and 42 days (P <0.05) compared to pre-immune rabbit sera (p>0.05) (**Figure 1C**). The antibody titer was found to be higher and of no or less reactivity for pre-immune rabbit serum. The graph was plotted for the obtained OD values against different dilutions from 1:800 to 1:12800 (**Figure 1D**).

### Immunoscreening of the *E. tarda* ED–BDU1 Gene Expression Library

**Figure 2:**
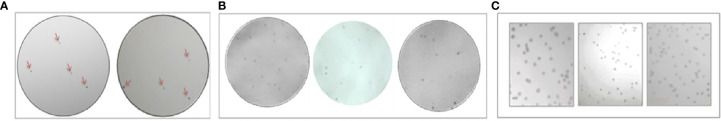
DNA library screening against rabbit polyclonal antiserum. **(A)** Primary screening in duplicates, arrow mark indicates the prominent spots developed. The positive plaques appeared as “doughnuts” with clear centers, **(B)** Secondary screening and **(C)** Tertiary screening (ED-BDU1 λ 1-1 III, ED-BDU1 λ 3-1 III, and ED-BDU1 λ 5-1 III).

A λ ZAPII library containing 2-5 kb random fragments of *E. tarda* was screened to identify phage that expressed gene products reactive with polyclonal rabbit sera. Primary screening was done with a titer of 5 X 10^4^ pfu and 600 µl of XL1-MRF cells at OD 600 of 0.5. Primary screening of approximately 10^5^ plaques of the lambda library against pooled rabbit hyperimmune serum revealed around 20 reactive plaques, which were further preceded for secondary screening (**Figure 2A, B**). Secondary screening shows 7 clones as hyper-reactive and others as less immunogen. Therefore the 7 clones that showed as positive were subjected to tertiary screening to identify the plaques showing 100% immunoreactivity frequency in the NC membrane. The tertiary screening identified 3 highly expressive immunoreactive clones namely BDU 1 λ 1-3 III, BDU 1 λ 3-1 III, and BDU 1 λ 5-1 III (**Figure 2C**). The ExAssist helper phage with SOLR strain (Strategene, San Diego, CA) was used to allow efficient excision of the pBluescript phagemid from the Lambda ZAP II vector.

### Obtaining of the Sequence of Antigenic Protein Gene Encoders for *E. tarda*

Plasmids were excised from the highly immunoreactive ED-BDU1+ λ recombinant phages (ED-BDU1 λ 1-3 III, ED-BDU1 λ 3-1 III, and ED-BDU1 λ 5-1 III) with the aid of ExAssist helper phage and *E.coli* SOLR. Insert DNAs were sequenced by using standard T7 and T3 primers. Sequence results found that the ED-BDU1 λ 1-3 III has two open reading frames, the first encodes a protein *hflK*, and the second encodes a protein *hflC*. Whereas the second clone (ED-BDU1 λ 3-1 III) encodes *crcA*. The third clone (ED-BDU1 λ 5-1 III) encodes the *yhcI.* The genetic sequencing analysis was performed by comparing the positive clones of *E. tarda* with the GenBank (**Table 3**).

**Table 3:** Genetic sequencing analysis by comparing the positive clones of *E. tarda* with the Gen Bank.

Clones	Genes	E-value^a^	Identity (%)	Region of the genome (Total gene)
ED-BDU1 λ 1-3 III	*hflK*,	0	100	399,238-400,527
	*hflC*	0	100	400,527-401,531
ED-BDU1 λ 3-1 III	*crcA*	0	100	2,823,483-2,823,956
ED-BDU 1 λ 5-1 III	*yhcI*	1e-07	97%	1,493,555-1,494,424

^a^The closer to zero the E-value, the lower the probability of the alignment of the gene sequences occurring at random.

### Cloning, Expression, and Immunoreactivity

**Figure 3:**
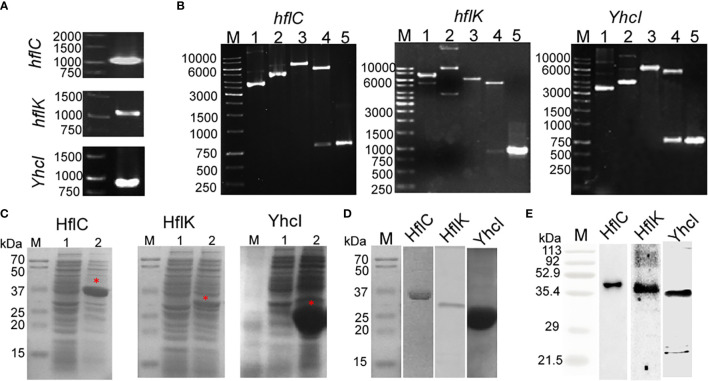
**(A)** PCR amplified product of hflC (a), hflK (b), and YhcI (c) from the genomic DNA of strain *E. tarda* ED-BDU1, **(B)** Restriction analysis and colony PCR analysis of hflC (a), hflK (b), and YhcI (c) from the *E. coli* BL21 transformants. (M- 1kb marker, 1- Vector pET28a, 2- construct pET28a+amplified gene, 3- single digestion of construct pET 28a+ amplified gene using EcoRI for hflC and hflK genes, BamHI for YhcI gene, 4-Double digestion of construct pET28a+ amplified gene using EcoRI and HindIII for hflC and hflK, BamHI genes and XhoI for YhcI gene and 5- Colony PCR product of the respective gene). **(C)** Expression of the hflC (a), hflK (b), and YhcI (c) proteins as confirmed by SDS-PAGE profiling. (Lane M: Marker, Lane 1: Uninduced whole cell lysate, Lane 2: IPTG induced whole cell lysate). **(D)** SDS- PAGE profiling of purified recombinant proteins (HflC, HflK, and YhcI). **(E)** Immunoblot analysis of purified recombinant proteins (HflC, HflK, and YhcI) probed with specific rabbit polyclonal antiserum.

**Figure 3A** shows that the PCR amplified product of the *hflC, hflK*, and *yhcI* from the genomic DNA of ED-BDU1 and were found to be1000 bp, 1100 bp, and 850 bp respectively. **Figure 3B** shows the REA and colony PCR analysis of *hflC, hflK*, and *yhcI* which reveals that the presence of the right insert. The expression of recombinant proteins (HflC, HflK, and YhcI) was confirmed by SDS- PAGE, and the molecular weight was found to be 36kDa, 40kDa, and 31kDa respectively (**Figure 3C**). The SDS profiling of purified proteins (**Figure 3D**) and the purified proteins were immunoblotted with rabbit’s sera and the immunoreactivity of the purified proteins was found to be specific (**Figure 3E**).

### Humoral Immune Response

**Figure 4:**
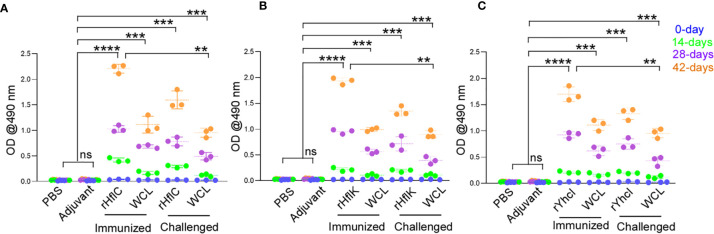
Evaluation of humoral immune response in control and immunized mice groups by ELISA. **(A)** rHflC antigen used, **(B)** rHflK antigen used and **(C)** rYhcI antigen used. Graphs represent the mean + SD of the optical density sera obtained at different day intervals (0, 14, 28, 42 days before challenge) and after challenge. ns, no significant, **P < 0.05, ***P < 0.01, ****P < 0.0001. P values were obtained by comparison with control (PBS and Adjuvant) using Tukey’s multiple comparisons test by 2-way ANOVA. (WCL- Whole cell lysate of *E. tarda*).

The specific reactivity of the serum samples against the recombinant HflK, HflC, and YhcI proteins was analyzed by IgG ELISA (**Figures 4A**–**C**). After the 21^st^ and 42^nd^ day of immunization, the significant induction of antibody titer was observed against the respective recombinant proteins in comparison with the negative control. There were significant levels of circulating anti-HflK, HflC, and YhcI were detected (P<0.0001). The developed sera were highly selective to the recombinant proteins ruling out the possibility of immunization. Similarly, the vaccinated groups show significant IgG response (P<0.0001), but lower compared to immunized alone.

### Cytokine Expression Profile

**Figure 5:**
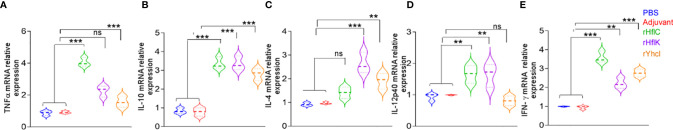
Cytokine profiling of the control and immunized mice. **(A)** TNFα, **(B)** IL-10, **(C)** IL-4, **(D)** IL-12p40, **(E)** IFN-γ. The relative CT (ΔΔ CT) method was used to quantify cytokine gene expression: CTs were normalized against the GAPDH gene CT (ΔCT) and then compared to the same normalized gene in the PBS immunized mice group. The control groups were set to 1. ns, no significance, **P < 0.01, ***P < 0.001. x-axis: Study groups, y-axis- Relative gene expression (fold).

mRNA abundance of both Th1 and Th2 type cytokines was evaluated by qRT-PCR analysis. Our results clearly show that our recombinant protein-based vaccines could induce significant levels of both Th1 and Th2 cytokines (p<0.05) (**Figure 5**). TNF- α (**Figure 5A**) mRNA expression levels were found to be upregulated in immunized groups like rHflC group (ratio = 4.05), rYhcI group (ratio = 1.56), and rHflK group (ratio = 2.26). IL10 (**Figure 5B**) mRNA expression was found to be significantly upregulated in HflC group (ratio = 3.35), YhcI group (ratio = 2.8) and HflK group (ratio = 3.34). IL-4 (**Figure 5C**) mRNA expression levels were found to be upregulated in the immunized HflK group (ratio =2.63) and YhcI group (ratio = 1.91) whereas others has less significant upregulation in the HflC group (ratio = 1.41). IL-12p40 (**Figure 5D**) mRNA expression levels were found to downregulated in YhcI group (ratio = 0.805) and the remaining groups HflC group (ratio = 1.655) and HflK group (ratio = 1.64) have significant levels of expression. IFN-γ (**Figure 5E**) mRNA expression levels were found to be upregulated in the HflC group (ratio = 3.57), YhcI group (ratio = 2.75) and HflK group (ratio = 2.22). Overall significant changes in mRNA expression levels were found to be present only in the HflC vaccine group for both the Th1 and Th2 type cytokines. Particularly, HflC based recombinant protein vaccine shows a good level of cytokine induction.

### Protective Efficacy of Vaccines

**Figure 6:**
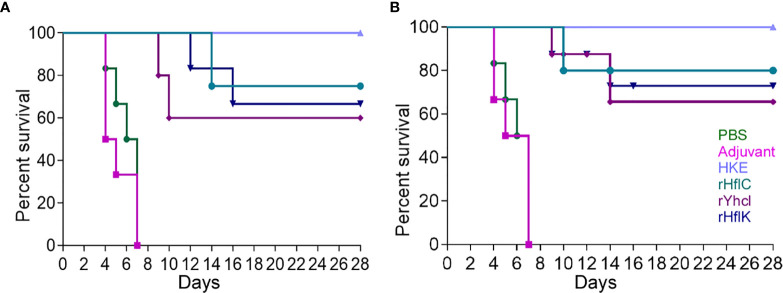
Survivability of the immunized mice **(A)** and fish **(B)** (*Labeo rohita*) against virulent *E. tarda* strain ED-BDU1 and the survival curves were compared using log-rank analysis. The number of survived animals in different day intervals (0–28) after the challenge was estimated as Mean ± SD to plot the graphs. Two-tailed P value was determined by Fisher exact test in comparison to the result of different groups observed as follows: PBS, Adjuvant, Heat Killed *Edwardsiella* (HKE), rHflC, rHflK, rYhcI. x-axis: Immunization days, y-axis: percent survival.

The LD50 dose was in the range of 1.6×10^5^ c.f.u. The protective efficacy of HflC, HflK, and YhcI was evaluated in terms of survival and reisolation of *Edwardsiella.* The survival data on the 30 days post-infection showed (60-100%) survival of animals in the vaccinated group (P <0.05), while none of the animals survived in the non-vaccinated group either PBS or Adjuvant (P <0.05). The rate of death of animals in PBS or adjuvant control (median survival time, 5 days) occurred immediately after the challenge confirming the virulence of the low passaged strain used for challenge experiments (**Figures 6A, B**).

## Discussion

The advantage of building an immune expressive library is that prior knowledge of target antibodies is not required, eliminating the need to design primer oligonucleotides, and allowing the identification of new antigens. Moreover, with a large part of the bacterium’s genome used in the library, it is possible to screen for the expression of several genes in the same experiment (20). In this study, immunoscreening of the gene expression library was efficient at finding genes that encode antigenic proteins of *E. tarda.*

The genes of *hflK* and *hflC* are adjacent to each other in the same operon and both having a single transmembrane segment located at the N-terminal region, form a complex (HflKC), remaining large C-terminal domain exposed on the periplasmic side. Also, evidence suggests that HflKC was inhibitory against the Sec-Y degrading function of FtsH both *in vivo* and *in vitro.* HflC (~45.5 kDa) and HflK (~38 kDa) help govern the stability of phage lambda cII protein, and thereby control the lysogenization frequency of phage lambda (21–24). Akiyama et al. proposed that HflC contains a serine–protease-like sequence motif. HflKC also forms a complex with FtsH, and represses the proteolytic activity of FtsH (25). HflKC, having an evolutionarily conserved domain (protein homology domain), may have protein/lipid-binding properties (26). HflK and HflC form a large complex with FtsH protease, that contributes to both membrane protein quality control and regulation of the cellular response to environmental stress in many bacteria. Both activities are crucial to the Lyme disease pathogen *Borrelia burgdorferi*, which depends on membrane functions, such as motility, protein transport, and cell signaling, to respond to rapid changes in its environment (27). In *Pseudomonas aeruginosa*, insertions inactivation of two FtsH protease accessory factors (HflK and HflC) and a cytoplasmic protease (HslUV) increased tobramycin sensitivity (28). YhcI encodes N-acetylmannosamine kinase [ManNac kinase (nanK)] which catalyzes the phosphorylation ManNAc to ManNAc-6-P liberated from N-acetylneuraminic acid (NANA) by NanA protein. Titgemeyer et al. (29) reported that YhcI is homologous to NagC, Mlc, and proteins of the ROK (repressor, ORF, and kinase) family. YhcI is also homologous to the bifunctional UDp-GlcNAc 2-epimerase/ManNAc kinase from the mammalian liver (30, 31).

Subunit vaccines are found to be safe and have negligible adverse effects when introduced in the form of purified recombinant proteins and formulated with certain adjuvants, which can induce the production of serum antibodies and produce highly protective effects (32, 33). In the present work, the recombinant proteins (rHflC, rHflK, and rYhcI) with 100 µg concentrations were injected into mice along with adjuvant (Alhydrogel). During the 4-week observation period, no mortality was found in the heat-killed *Edwardsiella* group when compared to rHflC (median survival: 14 days), rHflK (median survival: 16 days), rYhcI (median survival: 10 days), PBS (median survival: 6 days) and adjuvant alone group (median survival: 5 days). Nevertheless, the concentration used in the present study might not be the most optimal concentration, so further research is needed to optimize the concentration dose for challenges. A significant level of humoral immune response was detected in immunized animals with respective recombinant proteins. Moreover, the vaccinated animals showed significantly lower antibody titer than animals that underwent only immunization. Meanwhile, significant expression levels of Th1 and Th2 type cytokines were observed in the rHflC group compared to other groups. These findings relate that the rHflC could induce a strong humoral immune response.

In conclusion HflC, HflK and YhcI were identified as *in-vivo* expressed immunogenic proteins, which could induce a strong immune response upon infection and their recombinant proteins evoke a highly protective effect against *E. tarda* challenge. Together recombinant HflC elicits significant IL-10, IFN-γ, Th1, and Th2 mediated mRNA expression. This indicates that rHflC protein plays as a promising vaccine candidate against edwardsiellosis.

## Data Availability Statement

The datasets presented in this study can be found in online repositories. The names of the repository/repositories and accession number(s) can be found in the article/**Supplementary Material**.

## Ethics Statement

The animal study was reviewed and approved by Bharathidasan University Ethics Committee in Animal Experimentation.

## Author Contributions

PB: Conceptualization, methodology, software, and writing – original draft preparation. MG: Conceptualization, data curation, and methodology, Writing – original draft preparation. VV: Data curation and methodology. KA: Visualization and nvestigation. KP: Software, data curation, and methodology. NA-D: Supervision, editing, and funding acquisition. KN: Conceptualization, validation, funding acquisition, project administration, writing, and reviewing and editing. All authors contributed to the article and approved the submitted version.

## Funding

We acknowledge the Department of Biotechnology (DBT), the Government of India for the research and development grant (BT/PR12133/AAQ/3/707/2014 dated 02-09-2016) and the Researchers Supporting Project (RSP-2021/20) of King Saud University, Riyadh, Saudi Arabia.

## Conflict of Interest

The authors declare that the research was conducted in the absence of any commercial or financial relationships that could be construed as a potential conflict of interest.

## Publisher’s Note

All claims expressed in this article are solely those of the authors and do not necessarily represent those of their affiliated organizations, or those of the publisher, the editors and the reviewers. Any product that may be evaluated in this article, or claim that may be made by its manufacturer, is not guaranteed or endorsed by the publisher.
