# Supplementary material for: Construction of Genomic Library and Screening of Edwardsiella tarda Immunogenic Proteins for Their Protective Efficacy Against Edwardsiellosis
Source: Front Immunol. 2021 Nov 16;12:764662. doi: 10.3389/fimmu.2021.764662 (PMC8636194; doi:10.3389/fimmu.2021.764662)
Supplement: Supplementary file 1 [file DataSheet_1.docx]

**Supplementary data**

***In-vivo* expressed immunogenic proteins of *Edwardsiella tarda* and their protective efficacy against Edwardsiellosis**

Palanisamy Bothammal^1$^, Mohan Ganesh^1$^, Vellaisamy Vigneshwaran^1^, Kumarasamy Anbarasu^2^, Karuppiah Ponmurugan^3^, Naif Abdullah Al-Dhabib^3^, Kalimuthusamy Natarajaseenivasan^1^*

^1^Medical Microbiology Laboratory, Department of Microbiology, Center for Excellence in Life Sciences, Bharathidasan University, Tiruchirappalli – 620 024, Tamil Nadu, India

^2^Microbial Biotechnology Laboratory, Department of Marine Biotechnology, School of Marine Sciences, Bharathidasan University, Tiruchirappalli – 620 024, Tamil Nadu, India

^3^Department of Botany & Microbiology, College of Science, King Saud University, P.O.Box 2455, Riyadh – 11451, Kingdom of Saudi Arabia.

^$^Authors contributed equally

**Figure S1:** Genomic DNA **(A)** and 16S rRNA Gene Amplified product **(B)** of the *E. tarda* ATCC 15947 (lane 1) and the clinical isolates (lane 2- lane 14) (M- 1 kb DNA ladder).

**Table S1:** Study site, prevalence, and mortality among fishes due to edwardsiellosis

| **S. No.** | **Location** | **Mortality**  **(%)** | **No. of infected samples (n=27)** |
| --- | --- | --- | --- |
| 1 | Mattupatti | 18 | 3 |
| 2 | Vellanoor | 15 | 2 |
| 3 | Kalaikillainkudialloor | 20 | 4 |
| 4 | Aalambadi | 12 | 2 |
| 5 | Valadi | 18 | 3 |
| 6 | Koogunoor | 15 | 2 |
| 7 | Pambarampatti | 20 | 3 |
| 8 | Thirukampoor | 15 | 2 |
| 9 | Thuvaikudi | 18 | 2 |
| 10 | Manikandam | 25 | 4 |

**Table S2:** Biochemical Properties of ATCC 15947 and Clinical *E. tarda* Isolates

| ***E. tarda* strain** | **Sample Source** | **G** | **M** | **IN** | **MR** | **VP** | **C** | **TSI** | **U** | **GIU** | **M** | **MA** | **L** | **S** | **CAT** | **A** |
| --- | --- | --- | --- | --- | --- | --- | --- | --- | --- | --- | --- | --- | --- | --- | --- | --- |
| **ATCC 15947** | Kidney | - | + | + | + | - | - | + | - | + | + | + | - | - | + | + |
| **Ed –bdu 1** | Kidney | - | + | + | + | - | - | + | - | + | + | + | - | - | + | + |
| **Ed -bdu 2** | Liver | - | + | + | + | - | - | + | - | + | + | + | - | - | + | + |
| **Ed -bdu 3** | Kidney | - | + | + | + | - | - | + | - | + | + | + | - | - | + | + |
| **Ed -bdu 4** | Kidney | - | + | + | + | - | - | + | - | + | + | + | - | + | + | - |
| **Ed -bdu 5** | Liver | - | + | + | + | - | - | + | - | + | + | + | - | - | + | - |
| **Ed -bdu 6** | Liver | - | + | + | + | - | - | + | - | + | + | + | - | - | + | + |
| **Ed -bdu 7** | Kidney | - | + | + | + | - | - | + | - | + | + | + | - | - | + | - |
| **Ed -bdu 8** | Liver | - | + | + | + | - | - | + | - | + | + | + | - | - | + | - |
| **Ed -bdu 9** | Kidney | - | + | + | + | - | - | + | - | + | + | + | - | - | + | + |
| **Ed -bdu 10** | Kidney | - | + | + | + | - | - | + | - | + | + | + | - | - | + | + |
| **Ed -bdu 11** | Kidney | - | + | + | + | - | - | + | - | + | + | + | - | + | + | - |
| **Ed -bdu 12** | Kidney | - | + | + | + | - | - | + | - | + | + | + | - | - | + | ­ - |
| **Ed -bdu 13** | Kidney | - | + | + | + | - | - | + | - | + | + | + | - | - | + | + |

(+ Positive, - Negative, G – Gram stain, M – motility, IN – indole, MR –methyl red, VP –Voges Proskauer, C – citrate, TSI ­– triple sugar iron, U – urease, GLU – glucose, M – mannitol, MA – mannose, L –lactose, S ­– sucrose, CAT – catalase, A – arabinose)
